# Thrombospondin-1 Production Is Enhanced by *Porphyromonas gingivalis* Lipopolysaccharide in THP-1 Cells

**DOI:** 10.1371/journal.pone.0115107

**Published:** 2014-12-12

**Authors:** Misa Gokyu, Hiroaki Kobayashi, Hiromi Nanbara, Takeaki Sudo, Yuichi Ikeda, Tomonari Suda, Yuichi Izumi

**Affiliations:** Periodontology, Bio-Matrix Department, Graduate School of Medical and Dental Sciences, Tokyo Medical and Dental University, Tokyo, Japan; Charité, Campus Benjamin Franklin, Germany

## Abstract

Periodontitis is a chronic inflammatory disease caused by gram-negative anaerobic bacteria. Monocytes and macrophages stimulated by periodontopathic bacteria induce inflammatory mediators that cause tooth-supporting structure destruction and alveolar bone resorption. In this study, using a DNA microarray, we identified the enhanced gene expression of thrombospondin-1 (TSP-1) in human monocytic cells stimulated by *Porphyromonas gingivalis* lipopolysaccharide (LPS). TSP-1 is a multifunctional extracellular matrix protein that is upregulated during the inflammatory process. Recent studies have suggested that TSP-1 is associated with rheumatoid arthritis, diabetes mellitus, and osteoclastogenesis. TSP-1 is secreted from neutrophils, monocytes, and macrophages, which mediate immune responses at inflammatory regions. However, TSP-1 expression in periodontitis and the mechanisms underlying TSP-1 expression in human monocytic cells remain unknown. Here using real-time RT-PCR, we demonstrated that TSP-1 mRNA expression level was significantly upregulated in inflamed periodontitis gingival tissues and in *P. gingivalis* LPS-stimulated human monocytic cell line THP-1 cells. TSP-1 was expressed via Toll-like receptor (TLR) 2 and TLR4 pathways. In *P. gingivalis* LPS stimulation, TSP-1 expression was dependent upon TLR2 through the activation of NF-κB signaling. Furthermore, IL-17F synergistically enhanced *P. gingivalis* LPS-induced TSP-1 production. These results suggest that modulation of TSP-1 expression by *P. gingivalis* plays an important role in the progression and chronicity of periodontitis. It may also contribute a new target molecule for periodontal therapy.

## Introduction

Periodontitis is an inflammatory disease that is caused by gram-negative anaerobic periodontopathic bacteria, which colonize the tooth surface at marginal gingiva and the subgingival area [1]. Bacterial stimuli induce an immune response that produces a variety of pro-inflammatory cytokines and leads to the destruction of periodontal supportive tissue, resorption of the alveolar bone, and sometimes loss of the tooth [2]. *Porphyromonas gingivalis* is strongly associated with chronic periodontitis and is believed to play a crucial role in the disease process [3]. *P. gingivalis* possesses virulence factors such as fimbriae, gingipain, hemagglutinin, outer membrane proteins, lipopolysaccharide (LPS), and capsules [4], [5]. Of these, LPS is an important pathogenic component in the initiation and development of periodontitis [6]. Bacterial LPS promotes gingival inflammation with the increased expression of inflammatory cytokines and osteoclastogenesis activation that results in alveolar bone resorption.

Toll-like receptors (TLRs) on dendritic cells, monocytes, macrophages, and polymorphonuclear cells recognize invading stimuli such as LPS and promote activation of the innate immune system in tissues affected by periodontitis. In periodontal pockets, neutrophils are the dominant immune cells against bacteria. However, if neutrophils do not provide sufficient clearance, then bacterial penetration results in the activation of the monocyte/lymphocyte axis. The severity of disease progression is largely due to intrinsic differences in the monocyte/lymphocyte response traits [7]. During periodontal inflammation, monocytes play a key role in this immune response [8]. Human monocytes are exquisitely sensitive to LPS and respond by expressing inflammatory mediators. *P. gingivalis* LPS is reported to activate human monocytes [9] to induce inflammatory chemokines and cytokines such as IL-1α, IL-1β, IL-6, IL-10, TNF-α, CXCL10, and IL-32 [10], [11]. Thus, *P. gingivalis* LPS acts as an inflammatory signal through TLR pathways [12]. However, few studies have comprehensively examined the enhancement or suppression of the particular gene expression initiated by the host response against periodontal pathogens. An investigation to determine the numerous genes in the human monocytic cell line THP-1 cells stimulated by *P. gingivalis* LPS has not yet been conducted.

Thrombospondin-1 (TSP-1) is a 420–450-kDa homotrimeric multifunctional extracellular matrix protein, which was first isolated from human blood platelets as a thrombin-sensitive protein [13]. TSP-1 is secreted from endothelial cells, fibroblasts, neutrophils, monocytes, and macrophages [14]. Interacting with multiple different cell surface receptors, proteins, and proteoglycans of specific domains, TSP-1 has various but often converse biological effects [15]. TSP-1 is a regulator of TGF-β activation, which mediates wound healing, proliferation, cell differentiation, and cytokine responses [16]. TGF-β is a pleiotropic immunoregulatory cytokine found upregulated in periodontal gingival tissues [17]. TSP-1 stimulates macrophage migration, neutrophil phagocytosis, and monocyte chemotaxis to modulate the inflammatory response [18], [19]. It has an anti-inflammatory effect to inhibit NO activation and angiogenesis by binding CD36 [20], whereas it has been reported that CD47-TSP-1 interaction perpetuates the inflammation of rheumatoid synovitis [21]. TSP-1 promotes T-cell function by binding integrin or CD47 to regulate immune responses [21], [22]. Activated T-cells induces inflammatory cytokine production and receptor activator of nuclear factor-κB ligand (RANKL) expression, which causes local inflammation and bone destruction. Previous studies have shown that TSP-1 is abundantly expressed in damaged and inflamed tissues such as rheumatoid synovium, atherosclerotic lesions, and diabetes mellitus [18], [21]. TSP-1 deficiency decreases obesity-induced adipose tissue inflammation [19] and also attenuates experimental autoimmune encephalomyelitis [23]. Moreover, CD47-TSP-1 interaction is associated with RANKL-driven osteoclastogenesis and bone resorption [24]. These may be related to gingival inflammation and destruction of alveolar bone, which is characteristic of periodontitis. Although many reports have shown that TSP-1 is involved in the inflammation process, little is known about TSP-1 expression during periodontitis and the reaction of TSP-1 with *P. gingivalis* LPS.

The purpose of this study was to examine *P. gingivalis* LPS gene expression in human monocytic cells, to evaluate TSP-1 expression in periodontal gingival tissues, and to investigate the modulation of TSP-1 expression by periodontopathic bacteria. Here we show that *P. gingivalis* LPS induced TSP-1 expression in human monocytic cells. TSP-1 mRNA level was significantly increased in periodontitis gingival tissues. TSP-1 production was dependent upon TLR2/NF-κB signaling and was enhanced by T-cell cytokines. These results indicate that TSP-1 expression may play an important role in the progression of periodontitis.

## Materials and Methods

### Cell culture

A human acute monocytic leukemia cell line (THP-1) was obtained from ATCC (ATCC TIB-202, Rockville, MD, USA). THP-1 cells were cultured in RPMI-1640 with L-glutamine and phenol red (Wako, Osaka, Japan) supplemented with 10% fetal bovine serum (Gibco, Carlsbad, CA, USA) and 1% antibiotic–antimycotic penicillin–streptomycin (Invitrogen, Carlsbad, CA, USA). THP-1 cells were incubated in a 12-well culture plate (FALCON, Franklin Lakes, NJ, USA) at a concentration of 1.0×10^5^/ml. Cells were incubated at 37°C in a humidified atmosphere containing 5% CO_2_.

THP-1 cells were differentiated with phorbol 12-myristate 13-acetate (PMA) (Wako) at 25 ng/ml for 72 h. We removed the PMA containing media and incubated PMA-treated cells in RPMI-1640 for 24 h. PMA-treated cells were incubated at 37°C in a humidified atmosphere containing 5% CO_2_.

### Reagents

*P. gingivalis* LPS, Pam2CSK4, *Escherichia coli* (0111: B4 strain) LPS (InvivoGen, San Diego, CA, USA), IL-4, IL-17A, IL-17F, and IFN-γ (R&D SYSTEMS, Minneapolis, MN, USA) were used for stimulation. Pam2CSK4 and *E. coli* LPS were added to cell cultures at a final concentration of 1.0 µg/ml. IL-4, IL-17A, IL-17F, and IFN-γ were added at a final concentration of 10 ng/ml. IgG2a isotype control (BioLegend, San Diego, CA, USA), TLR2-neutralizing antibody (anti-TLR2), and TLR4-neutralizing antibody (anti-TLR4) (IMGENEX, San Diego, CA, USA) were used to block the biological activity of TLRs; they were added at a final concentration of 10 ng/ml 30 min prior to stimulation with *P. gingivalis* LPS, Pam2CSK4, and *E. coli* LPS. For *P. gingivalis* LPS stimulation, various doses of LPS (0.0001, 0.001, 0.01, 0.1, and 1.0 µg/ml) were added to the THP-1 cell and PMA-treated cell culture and stimulated for various time periods (1, 2, 4, 12, 24, 48, and 72 h). MG-132 (Calbiochem, Darmstadt, Germany), a specific inhibitor of NF-κB, was added at a final concentration of 5.0 ng/ml 1 h prior to stimulation with *P. gingivalis* LPS.

### Human gingival tissues

Fifteen human inflammatory gingival tissues were obtained during flap operations from deep periodontal pocket gingiva [probing pocket depth (PPD) ≥4 mm, bleeding of probing was positive], 13 inflammatory gingival tissues were obtained during apically positioned flap surgery from shallow periodontal pocket gingiva (PPD <4 mm, bleeding of probing was positive), and 7 healthy gingival grafts with no inflammation were obtained during free gingival graft operations from palatal gingival tissues. The samples of human gingival tissues were divided into 3 groups. Collected samples were immediately immersed in RNAlater (RNA Stabilization Reagent; QIAGEN, Hilden, Germany). Single tissue pieces were obtained from the tube and ground using FastPrep-24 and Lysing Matrix A (M.P. Biomedicals, Santa Ana, CA, USA). Samples were stored at −20°C.

This study was performed with the approval of the Ethics Committee of Tokyo Medical and Dental University (#879). All sampling was performed after obtaining written informed consent from the patient.

### DNA microarray

For gene expression study, mRNA was extracted from THP-1 cells treated with 1.0 µg/ml *P. gingivalis* LPS or untreated after 4 h [10]. Total RNA was electrophoresed using a 2100 Bioanalyzer (Agilent Technologies, Santa Clara, CA, USA) and measured for quality and concentration using Nano Drop (Thermo SCIENTIFIC, Waltham, MA, USA). The concentrations of *P. gingivalis* LPS stimulating sample (Test) and control sample (Control) were 141.9 ng/µl and 254.0 ng/µl, respectively. The ratio of optical density (OD) at 260 nm to OD at 280 nm (OD_260/280_) for both Test and Control was similar at 2.07. Each sample underwent RNA amplification using an Ambion Amino Allyl aRNA kit (#1753; Ambion, Carlsbad, CA, USA) using the steps of first and second strand cDNA synthesis, clean-up of double-strand cDNA, synthesis of aRNA, and clean-up of aRNA. The samples were checked by Nano Drop for aRNA amplification. Using 10 µl of the amplified RNA, the Test was labeled with Amersham Cyanine-5 Monoreactive Dye (Cy5; #PA25001; GE, Healthcare Life Sciences Pittsburgh, PA, USA) and the Control was labeled with Amersham Cyanine-3 Monoreactive Dye (Cy3; #PA23001; GE). Samples were purified (YM30/#42407; Microcon, Billerica, MA, USA), fragmented, and purified again (YM10/#42410, Microcon). To measure the Nano Drop labeling efficiency, each 1.0-µl sample was hybridized using Human Oligo chip 25K (TORAY, Tokyo, Japan) at 37°C for 16 h using a hybridization chamber (Takara-bio, #TX711, Otsu, Japan) and a bio-shaker. Following hybridization and washing, the chip was scanned and quantified using a 3D-Gene Scanner 3000 (TORAY). The raw data sheets show the BG subtraction value, which was obtained by subtracting the background value from the raw data, and the global normalization value of the normalized data, which was obtained by adjusting the median of the Cy3/Cy5 ratio to 1. This DNA array analysis was all performed by 3D-Gene (TORAY).

### RNA extraction and real time RT-PCR

Total RNA from cells and gingival tissues was extracted using an RNeasy Mini kit (QIAGEN) according to the manufacturer's instructions. RNA quantitation and purity were calculated by measuring the ratio of absorbance at 260 nm to the absorbance at 280 nm using NanoDrop Lite (Thermo Scientific). cDNA synthesis was conducted using a QuantiTect Reverse Transcription kit (QIAGEN). For each sample, 1.0 µg of total RNA was used for each 20-µl reverse transcription reaction system. The reaction mixture contained 12.5 µl of SYBR Premix Ex Taq II (Takara-bio), 0.8 µl of forward and reverse primers and 1.0 µl of sample cDNA. PCR reaction efficiency was optimized, and the final concentration of each primer was 0.4 µM. Real time RT-PCR was performed with a Thermal Cycler Dice Real Time System II (Takara-bio) with the following thermal profile: and sets of primers (Takara-bio):

One cycle at 95°C for 30 s, 40 cycles at 95°C for 5 s and 60°C for 30 s, and 1 cycle at 95°C for 15 s, 60°C for 30 s, and 95°C for 15 s.

TSP-1 Forward: 5′-ATG GAA TTG GTG ATG CCT GTG-3′,

TSP-1 Reverse: 5′-ACT GAG CTG GGT TGT AAT GGA ATG-3′,

GAPDH Forward: 5′-GCA CCG TCA AGG CTG AGA AC-3′, and

GAPDH Reverse: 5′-TGG TGA AGA CGC CAG TGG A-3′.

The relative quantification of mRNA was calculated using the Standard Curve Method. Threshold Cycle was based on the Crossing Point Method. Standard curves were made with control cDNA. The mRNA expression levels were normalized against GAPDH mRNA. All data were analyzed using Thermal Cycler Dice Real Time System Software (Takara-bio).

### ELISA

THP-1 cells were stimulated with 0, 0.01, 0.1, and 1.0 µg/ml *P. gingivalis* LPS for 4 h. THP-1 cells were stimulated with 1.0 µg/ml *P. gingivalis* LPS for 0, 1, 4, 6, 12, 24, 48, and 72 h. THP-1 supernatants were analyzed to determine the amount of TSP-1 using ELISA kits (Quantikine TSP-1 ELISA kit; R&D SYSTEMS) and following the manufacturer's instructions. OD was measured at a wavelength of 450 nm.

### Statistical analysis

All results are shown as means and standard deviations (SD). Data from triplicate samples were used for statistical analysis. The statistical significance of the results was calculated using the Tukey–Kramer multiple comparisons test by InStat (GraphPad Software, La Jolla, CA, USA). Differences were considered significant at *P*<0.05.

## Results

### TSP-1 mRNA expression was significantly upregulated in periodontitis gingival tissues

**Figure 1:**
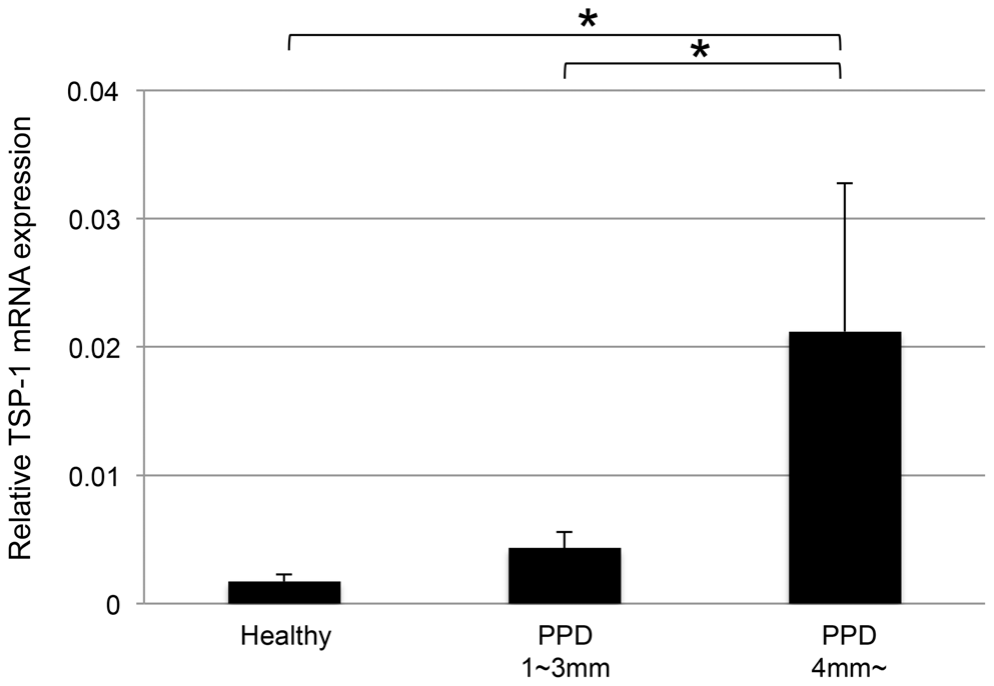
The level of TSP-1 mRNA expression was significantly upregulated in periodontitis gingival tissues. Human gingival tissues were obtained from periodontal surgical operations. TSP-1 mRNA expression was enhanced in inflammatory gingival tissues. Its expression was significantly higher in regions of deep periodontal pockets than in shallow periodontal pockets and healthy sites (*P*<0.001).

The level of TSP-1 mRNA expression was significantly upregulated in the deep periodontal pocket gingiva group (PPD ≥4 mm) compared with that in the shallow periodontal pocket gingiva (PPD <4 mm) and no inflammatory gingiva groups (Healthy; Fig. 1). PPD was 5.6±1.5 mm (mean ± SD) in the Deep periodontal pocket group and 2.4±0.5 mm in the Shallow periodontal pocket group. Between these two groups, a 2-tailed *P* value of <0.0001 was considered extremely significant. There was a tendency for increased production of TSP-1 mRNA as the periodontal pocket became deeper.

### *P. gingivalis* LPS induced different types of gene expression in THP-1 cells

3D-Gene microarray-based gene expression profiling was conducted using a gene chip focused on major molecules. THP-1 cells were stimulated with 1.0 µg/ml *P. gingivalis* LPS for 4 h and 1.0 µg of total RNA was used for detection. The results of this analysis are listed in S1 Table. The total data sheet shows the raw data values; the BG subtraction value, which was obtained by subtracting the background value from the raw data, and the global normalization value of normalized data, which was obtained by adjusting the median of the Cy3/Cy5 ratio to 1. In addition to known chemokines and cytokines, expression of a wide variety of genes, including that encoding TSP-1, was observed. A total of 25,369 genes were detected, of which an increased expression of 2-fold or more was found for 1974 genes, whereas 1247 genes were similarly downregulated. We focused on 134 genes whose expression was increased more than 10-fold. Of particular interest was TSP-1, a protein encoded by the *THBS1* gene; this was increased more than 14.5-fold.

### TSP-1 was expressed in response to *P. gingivalis* LPS by THP-1 cells

**Figure 2:**
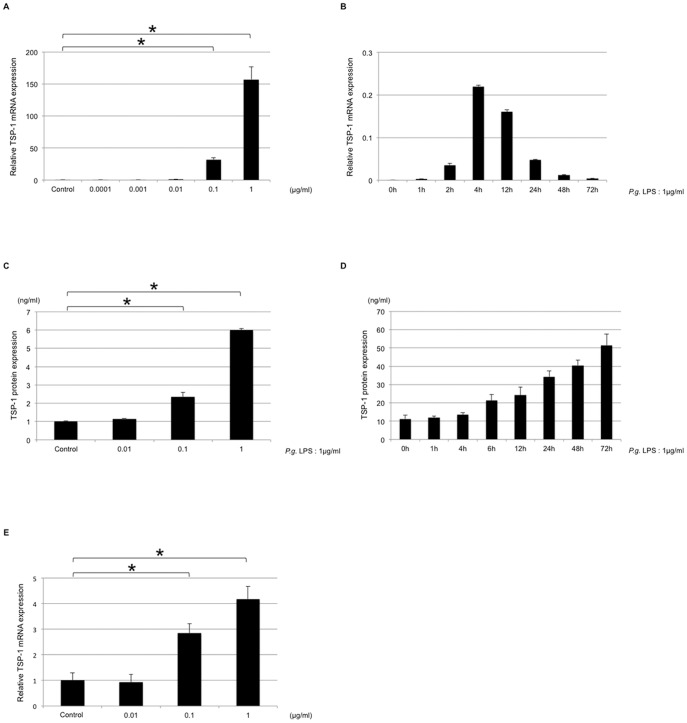
Upregulation of TSP-1 in THP-1 cells by *P. gingivalis* LPS stimulation. (A) THP-1 cells were stimulated by *P. gingivalis* LPS at concentrations of 0, 0.0001, 0.001, 0.01, 0.1, or 1.0 µg/ml for 4 h. *P. gingivalis* LPS increased TSP-1 mRNA expression in a dose-dependent manner in THP-1 cells. (B) THP-1 cells were stimulated by 1.0 µg/ml of *P. gingivalis* LPS for 0, 1, 2, 4, 12, 24, 48, or 72 h. *P. gingivalis* LPS increased TSP-1 mRNA expression in a time-dependent manner in THP-1 cells. (C) THP-1 cells were stimulated with *P. gingivalis* LPS at concentrations of 0, 0.01, 0.1, or 1.0 µg/ml for 72 h. *P. gingivalis* LPS increased TSP-1 protein production in a dose-dependent manner in THP-1 cells. (D) THP-1 cells were stimulated with 1.0 µg/ml of *P. gingivalis* LPS for 0, 1, 4, 6, 12, 24, 48, or 72 h. *P. gingivalis* LPS increased TSP-1 protein production in a time-dependent manner in THP-1 cells. (E) PMA-treated THP-1 cells were stimulated with *P. gingivalis* LPS at concentrations of 0, 0.01, 0.1, or 1.0 µg/ml for 72 h. *P. gingivalis* LPS increased TSP-1 protein production in a dose-dependent manner in PMA-treated THP-1 cells.

Fig. 2 demonstrates that TSP-1 expression was enhanced by *P. gingivalis* LPS in THP-1 cells. The highest expression was observed at a concentration of 1.0 µg/ml (Fig. 2A). TSP-1 mRNA expression at concentrations of 1.0 µg/ml and 0.1 µg/ml *P. gingivalis* LPS was significantly different from that observed without stimulation (*P*<0.05 and *P*<0.001, respectively). The highest expression was observed at 4 h of stimulation with the *P. gingivalis* LPS preparation (Fig. 2B). THP-1 cells with *P. gingivalis* LPS preparations reached a peak after 4 h; this corresponds to the fact that TSP-1 is transiently released early during the acute inflammatory phase. The secretion of TSP-1 protein was increased in a time and dose-dependent manner (Fig. 2C, 2D). TSP-1 mRNA expression was also upregulated in PMA-treated THP-1 cells stimulated with *P. gingivalis* LPS (Fig. 2E).

### *P. gingivalis* LPS induced TSP-1 expression via TLR2 by THP-1 cells

**Figure 3:**
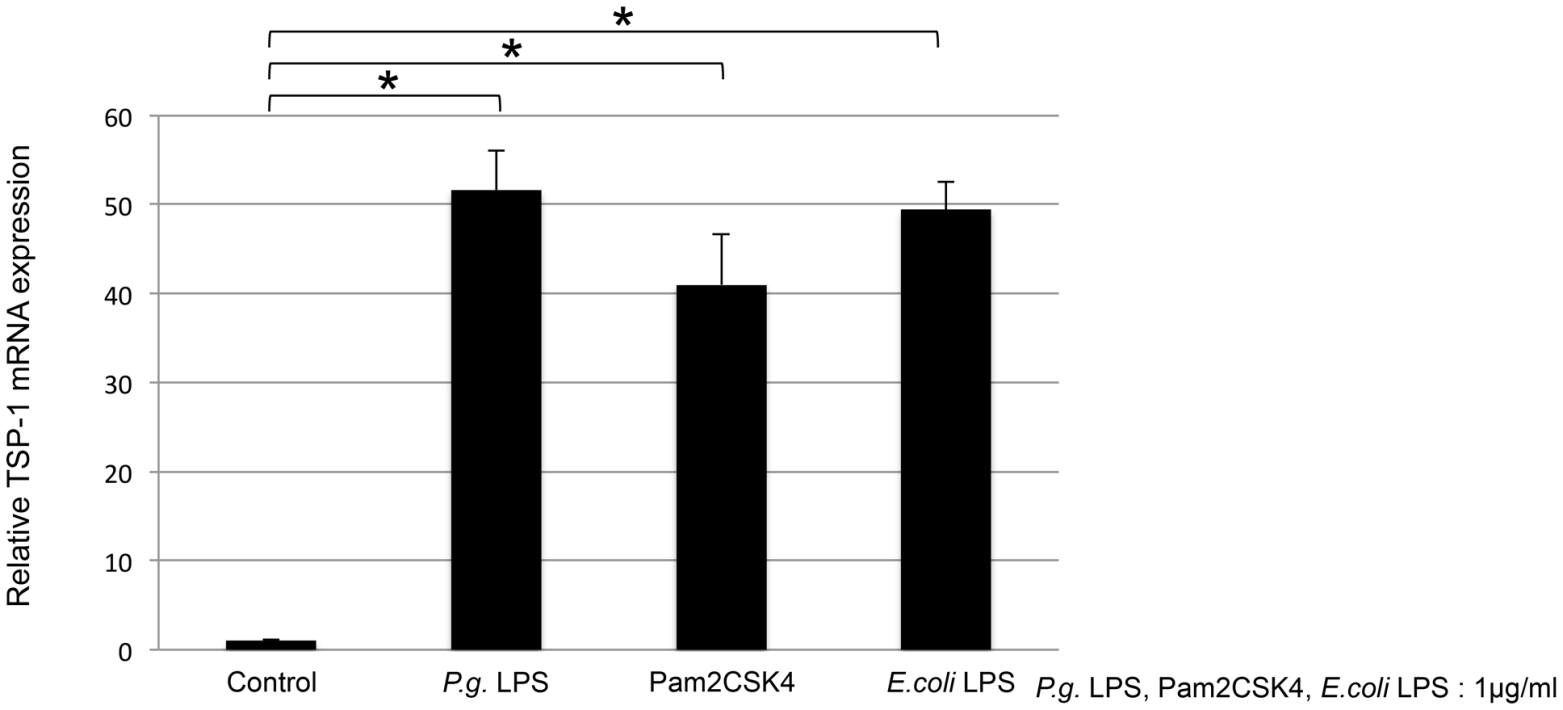
TSP-1 mRNA expression in THP-1 cells by *P. gingivalis* LPS, TLR2 ligand, or TLR4 ligand. THP-1 cells were stimulated with 1.0 µg/ml of *P. gingivalis* LPS, Pam2CSK4 (TLR2 ligand), or *E. coli* LPS (TLR4 ligand) for 4 h. *P. gingivalis* LPS, Pam2SCK4, and *E. coli* LPS significantly increased TSP-1 mRNA expression in THP-1 cells (*P*<0.001).

**Figure 4:**
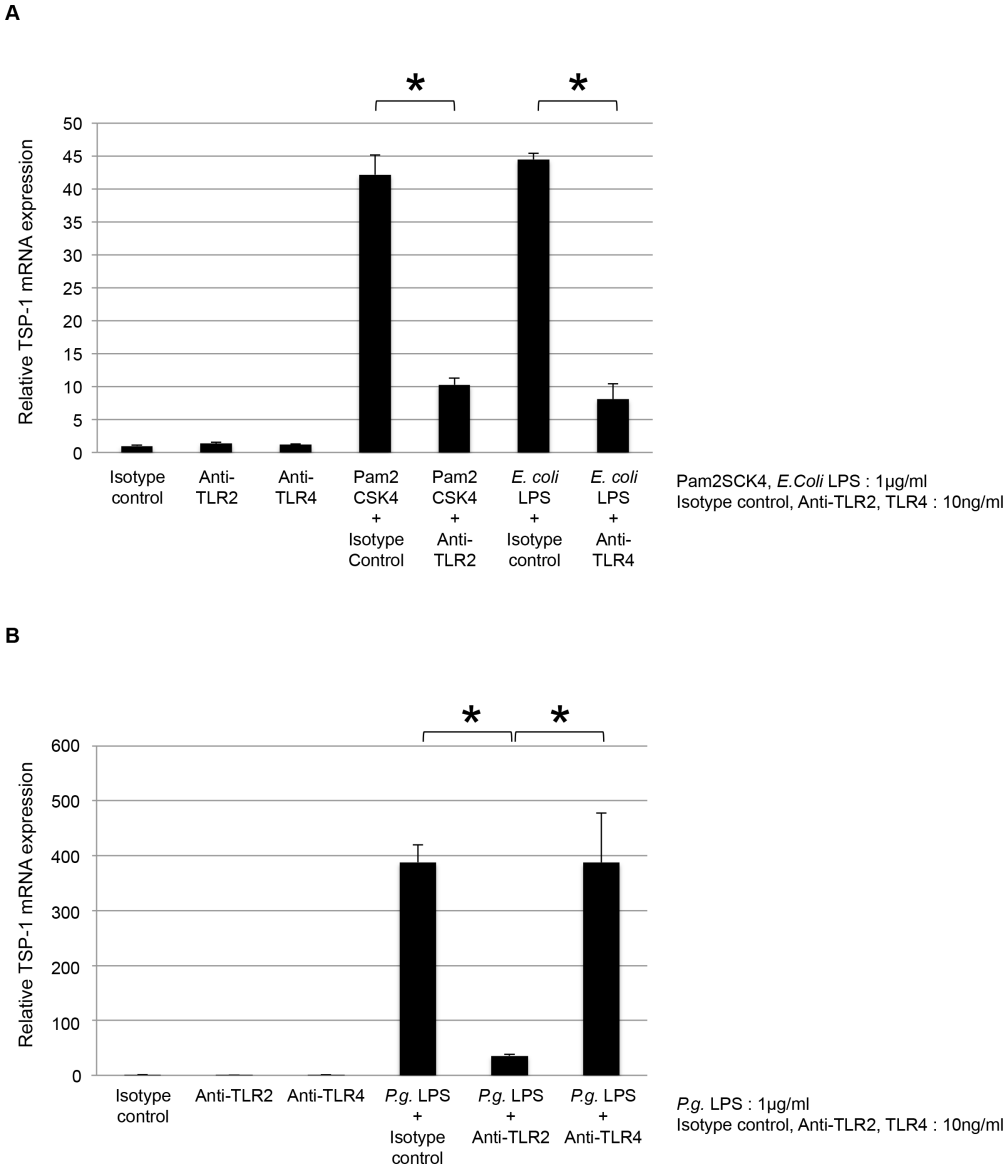
*P. gingivalis* LPS-induced TSP-1 mRNA expression was inhibited by TLR2-neutralizing antibody in THP-1 cells. THP-1 cells were pretreated with 10 ng/ml of IgG2a isotype control, TLR2-neutralizing antibody, and TLR4-neutralizing antibody for 30 min, followed by the addition of 1.0 µg/ml of *P. gingivalis* LPS, Pam2CSK4, and *E. coli* LPS for 4 h. (A) TLR2-neutralizing antibody and TLR4-neutralizing antibody neutralized the activity of Pam2CSK4 and *E. coli* LPS, respectively. (B) *P. gingivalis* LPS with TLR2-neutralizing antibody significantly reduced TSP-1 mRNA expression compared with *P. gingivalis* LPS alone in THP-1 cells (*P*<0.001).

To examine whether *P. gingivalis* LPS required TLR2 or TLR4, THP-1 cells were stimulated with 1.0 µg/ml of *P. gingivalis* LPS, Pam2CSK4 (TLR2 ligand), or *E. coli* LPS (TLR4 ligand) for 4 h. All 3 stimulatory factors significantly increased TSP-1 mRNA expression (*P*<0.001; Fig. 3). This result suggested that *P. gingivalis* LPS utilized both TLR2 and TLR4 pathways in this study. Furthermore, THP-1 cells were pretreated with 10 ng/ml of isotype control, anti-TLR2, and anti-TLR4 for 30 min, followed by treatment with 1.0 µg/ml of *P. gingivalis* LPS, Pam2CSK4, and *E. coli* LPS for 4 h. Anti-TLR2 and anti-TLR4 significantly inhibited TSP-1 mRNA expression by Pam2CSK4 and *E. coli* LPS, respectively (*P*<0.001; Fig. 4A). Anti-TLR2 significantly reduced *P. gingivalis* LPS-induced TSP-1 mRNA expression (*P*<0.001; Fig. 4B). Anti-TLR4 had little influence on expression (Fig. 4B). These data indicated that *P. gingivalis* LPS-induced TSP-1 expression was upregulated in THP-1 cells via the TLR2 pathway.

### TSP-1 expression was increased by co-stimulation with *P. gingivalis* LPS and IL-4, IL-17A, IL-17F, and IFN-γ

**Figure 5:**
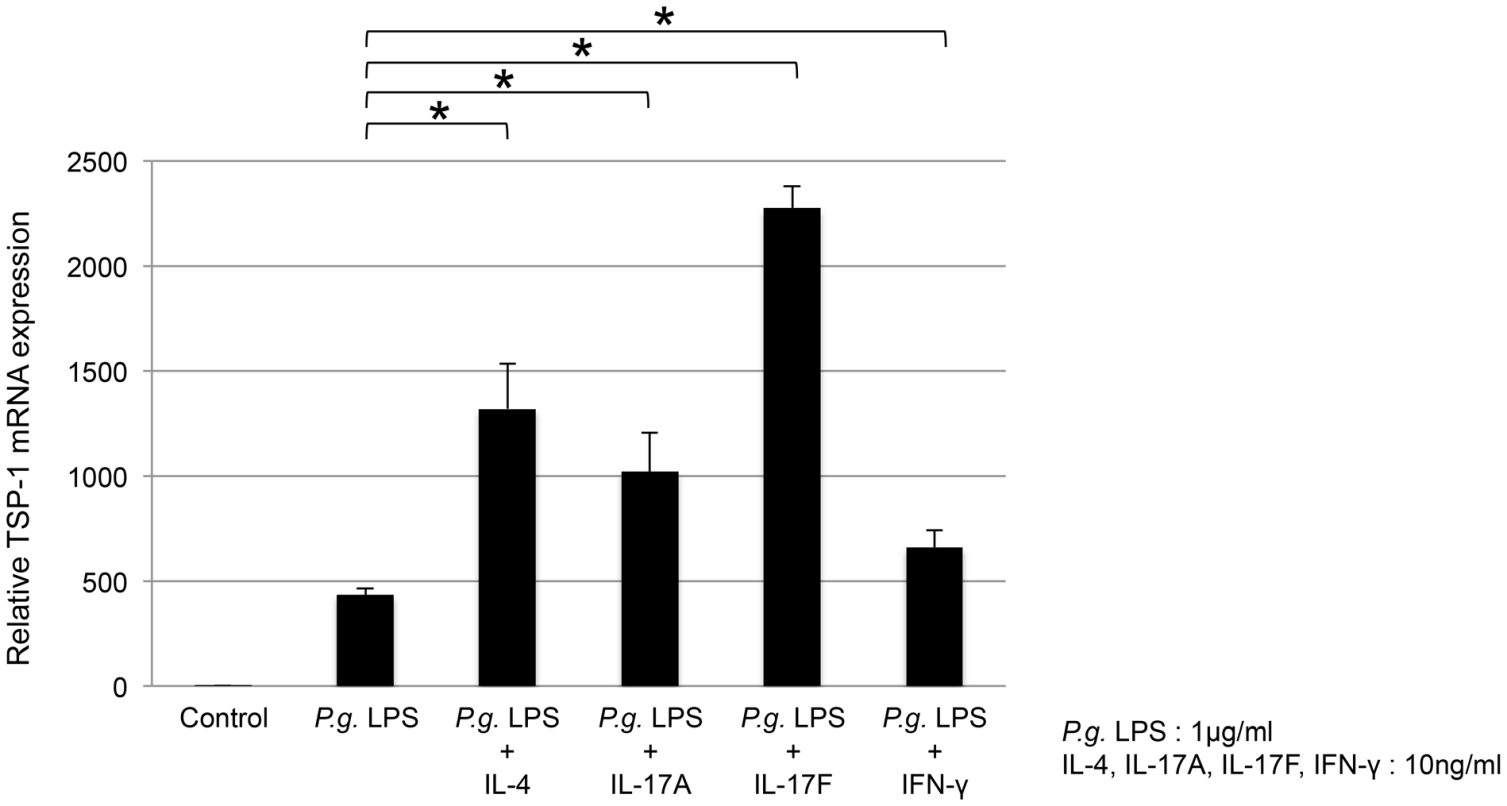
Effect of various cytokines on TSP-1 expression in THP-1 cells. THP-1 cells were co-stimulated with 1.0 µg/ml of *P. gingivalis* LPS and 10 ng/ml of each of IL-4, IL-17A, IL-17F, or IFN-γ for 4 h. TSP-1 mRNA expression was significantly enhanced by all co-stimulatory factors (*P*<0.001). In particular, TSP-1 mRNA expression by *P. gingivalis* LPS together with IL-17F was high.

THP-1 cells were co-stimulated with 1.0 µg/ml of *P. gingivalis* LPS and 10 ng/ml of each of IL-4, IL-17A, IL-17F, and IFN-γ for 4 h. TSP-1 mRNA expression was significantly enhanced by IL-4, IL-17A, IL-17F, and IFN-γ (*P*<0.001; Fig. 5). In particular, TSP-1 mRNA expression induced by *P. gingivalis* LPS together with IL-17F was more than 5 times higher than that induced by *P. gingivalis* LPS alone. Each cytokine synergistically enhanced *P. gingivalis* LPS-induced TSP-1 production. In THP-1 cells stimulated with *P. gingivalis* LPS, IL-4, IL-17A, IL-17F, and IFN-γ had an effect of promoting TSP-1 mRNA expression.

### *P. gingivalis* LPS-induced TSP-1 expression in THP-1 cells was dependent upon NF-κB signaling

**Figure 6:**
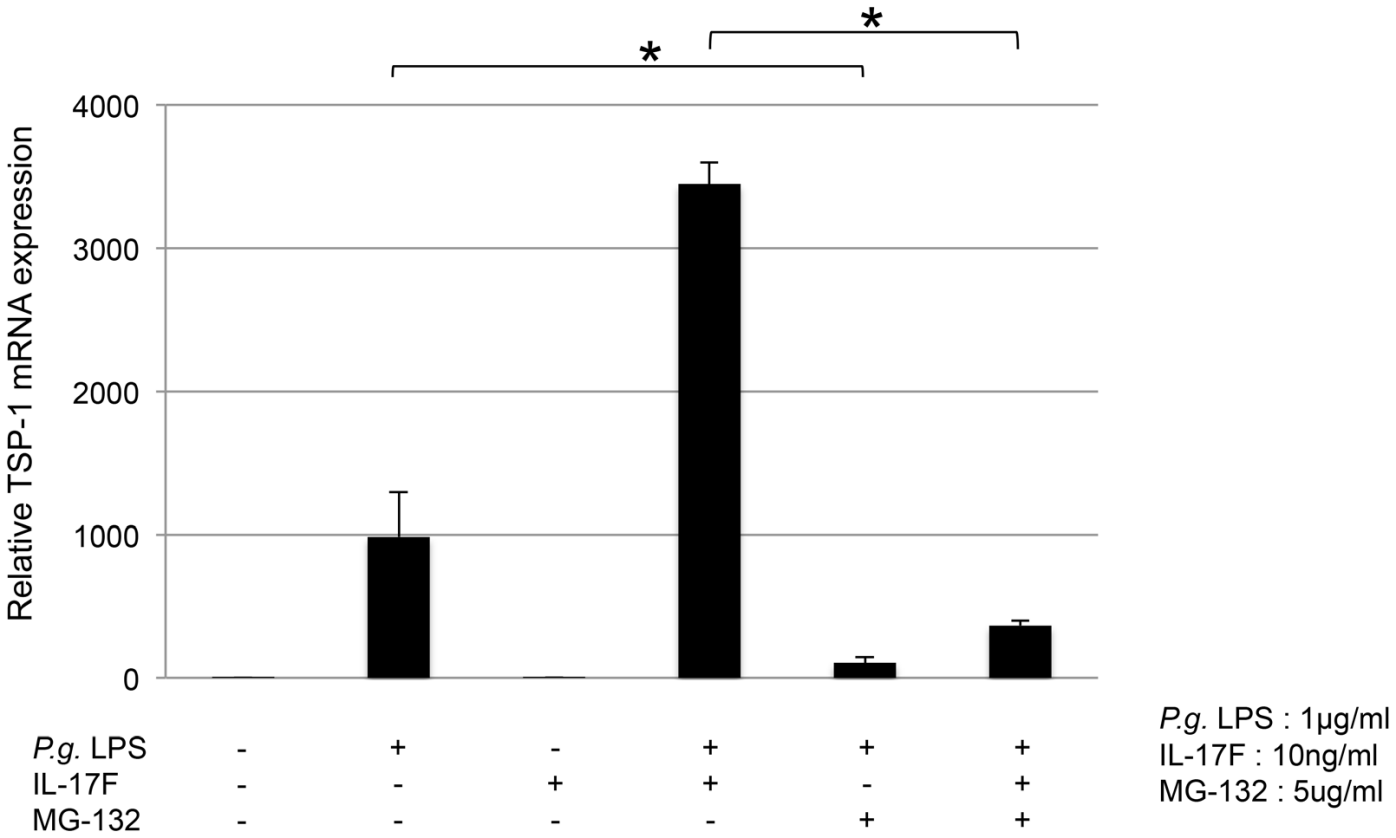
MG-132 inhibited TSP-1 mRNA expression in THP-1 cells. THP-1 cells were pretreated with 5.0 ng/ml of MG-132 (inhibitor of NF-κB) for 1 h, followed by the addition of 1.0 µg/ml of *P. gingivalis* LPS and 10 ng/ml of IL-17F. *P. gingivalis* LPS-induced TSP-1 mRNA was significantly reduced by MG-132. MG-132 also significantly reduced TSP-1 mRNA expression induced by *P. gingivalis* LPS together with IL-17F.

THP-1 cells were pretreated with 5.0 ng/ml of MG-132 (an inhibitor of NF-κB) for 1 h, followed by the addition of 1.0 µg/ml of *P. gingivalis* LPS and 10 ng/ml of IL-17F. MG-132 significantly reduced *P. gingivalis* LPS-induced TSP-1 mRNA expression (Fig. 6). MG-132 also significantly reduced TSP-1 mRNA expression by co-stimulation with *P. gingivalis* LPS and IL-17F (Fig. 6). However, IL-17F stimulation alone had no effect on TSP-1 mRNA production. Therefore, *P. gingivalis* LPS-induced TSP-1 mRNA expression in THP-1 cells was dependent upon NF-κB signaling.

## Discussion

This study investigated the modulation of TSP-1 expression by periodontal pathogenic factor *P. gingivalis* LPS. Our study revealed that TSP-1 mRNA was significantly upregulated in severe periodontitis gingival tissues. *P. gingivalis* LPS induced TSP-1 expression in human monocytic cells through TLR2/NF-κB signaling; this TSP-1 production was also enhanced by inflammatory cytokines such as IL-17F.

Here we focused on TSP-1 from the results of a 3D-Gene microarray. Many well-known genes were identified in the array results; among them, we particularly focused on TSP-1, which has not been previously reported in periodontitis. In this study, high levels of TSP-1 mRNA expression were observed in human inflammatory gingival tissues; however, TSP-1 mRNA expression was hardly observed in healthy tissues in the oral cavity. Inflammatory cytokines such as IL-1β, TNF-α, IFN-γ, and IL- 2 were observed in inflamed gingival tissue, which had deep probing pocket depths [25]. TSP-1 mRNA expression was also significantly enhanced in deep periodontal pocket sites where gingival tissue destruction was severe. *P. gingivalis* has an ability to attach and invade periodontium cells, and significant correlation was observed between the periodontal pocket depth and the degree of *P. gingivalis* invasion [26]. In this study, *P. gingivalis* LPS acted in a dose-dependent manner on TSP-1 mRNA and protein expression in *in vitro* experiments, suggesting that high amounts of *P. gingivalis* have the potential to increase TSP-1 in inflammatory gingival tissues.

In some previous studies, large amounts of TSP-1 were expressed at the sites of damaged and inflamed tissues. TSP-1 expression was upregulated in rheumatoid synovial inflammatory tissues [21], [27]. Increased expression of TSP-1 facilitated T-cell infiltration, which resulted in persistent tissue damage in inflamed joints [21]. TSP-1 mRNA expression was upregulated in myositis muscles to perpetuate the inflammatory response [28]. Periodontopathic bacterial infection is believed to activate RANKL expression by T-cells. TSP-1 produced by *P. gingivalis* LPS may be involved in periodontal inflammation and RANKL-driven bone resorption. Therefore, TSP-1 expression may be associated with the severity and chronicity of periodontitis.

However, other studies indicate different inflammatory effects of TSP-1. TSP-1-deficient mice had persistent pneumonia because of a defect in the regulation of inflammatory cells [29]. TSP-1 peptide decreased the inflammation of experimental erosive arthritis by reducing granulocytosis [30]. In an experimental autoimmune uveoretinitis model, TSP-1 knockout mice developed severe diseases [23]. These findings suggest that TSP-1 has both anti-inflammatory and pro-inflammatory mechanisms. TSP-1 interacts with multiple receptors such as CD36, CD47, and integrins [20], suggesting that TSP-1 has contrasting functions. CD36 has been reported to be abundantly co-expressed with TSP-1 in rheumatoid synovium macrophages and endothelial cells [31]. TSP-1, CD36, and CD47 expression have been reported to be upregulated in inclusion body myositis and dermatomyositis muscle specimens [28]. TSP-1 has a strong anti-angiogenic function, which is believed to impact chronic inflammation. These results indicate that TSP-1 may have some role in periodontal inflammation and alveolar bone resorption.

This study was undertaken to investigate the modulation of TSP-1 expression in periodontitis through the stimulation of human monocytic cell line THP-1 cells by *P. gingivalis* LPS. The present study shows that not only *P. gingivalis* LPS but also Pam2CSK4 and *E. coli* LPS promote TSP-1 expression in THP-1 cells. Immunocompetent cells such as macrophages, monocytes, and dendritic cells and periodontal tissue component cells such as gingival epithelial cells and fibroblasts expressed TLR on their cell surfaces. Previous studies have shown that TLR2 and TLR4 are expressed on periodontium cells [32]; in particular, TLR2 and TLR4 are deeply involved in the immune response of periodontal tissues [33]. In a previous study, TLR2 and TLR4 mRNA were expressed at significant levels in THP-1 cells, and TLR2 expression appeared to be stronger than TLR4 expression [34]. In our study, TSP-1 was upregulated by stimulation with the *P. gingivalis* LPS preparation. Its expression was inhibited by anti-TLR2 but remained unchanged with anti-TLR4, although the LPS of gram-negative bacteria was normally recognized by TLR4 [35]. There is a report that *E. coli* LPS-stimulated bone marrow-derived macrophages enhance TSP-1 production via TLR4 [36]. On the other hand, *P. gingivalis* LPS is less dependent on TLR4 signaling than *E. coli* LPS [37]. *P. gingivalis* LPS can be utilized through both TLR2 and TLR4 with its unique lipid A structure [12]. In mouse models, TLR2-deficient mice resisted alveolar bone loss following oral infection with *P. gingivalis* [38], although another study showed that TLR4, not TLR2, was important in protecting against alveolar bone loss [39]. TLR2 plays a sentinel role in the defense against microbial pathogens in the innate immune response [40], [41]. It is believed that TSP-1 is produced through TLR2 as well as TLR4 in THP-1 cells by *P. gingivalis* LPS, and TLR2 is a primary pathway for *P. gingivalis* LPS, which leads to TSP-1 expression in THP-1 cells. Moreover, its expression is dependent on the NF-κB pathway downstream of TLR2.

The present findings showed that TSP-1 production by *P. gingivalis* LPS was enhanced by inflammatory cytokines. This is consistent with previous findings where high plasma levels of TSP-1 were correlated with CCL4/MIP1β and TGF-β in patients with rheumatoid arthritis [42]. TNF-α upregulates TSP-1 production in human skeletal muscle-derived cell line myoblasts [28]. Furthermore, in periodontitis, there is a possibility that TSP-1 co-stimulatory with various cytokines activates T-cells or other immune cells. A previous study demonstrated that the addition of Th1 and Th2 cytokines reduced *E. coli* LPS-mediated TSP-1 production, whereas IL-17 had no effect on bone marrow-derived macrophages [36]. In our study, co-stimulation with *P. gingivalis* LPS and either IL-4, IFN-γ, IL-17A, or particularly IL-17F upregulated TSP-1 expression in THP-1 cells. TSP-1 is closely associated with Th17 differentiation through its ability to activate latent TGF-β [23]. It is therefore suggested that THP-1 cells can continue to secrete TSP-1 in a Th17 environment. Innate immune cells such as neutrophils also produce IL-17 cytokine at an early phase of stimulation [43], [44]. A large amount of IL-17 is observed in *P. gingivalis* oral injection model mice [44]. High amounts of IL-17 in periodontal regions have been reported, and *P. gingivalis* has been associated with IL-17 levels [45]. *P. gingivalis* and IL-17 work in tandem in TSP-1 production. However, TSP-1 expression was not increased by IL-17F alone. Although further studies are needed, IL-17F may exhibit potential interaction through the downstream signaling of TLR2. Through the interaction with various cytokines, TSP-1 may have a synergistic effect on aggravated inflammation. It may be useful for the development of treatment and new prevention. In some cases, the inflammatory response that should protect against infection promotes host's tissue destruction. It is necessary to note the disease treatment strategy.

Our study demonstrated that TSP-1 is elevated in periodontal gingival tissues and TSP-1 expression is induced by *P. gingivalis* LPS stimuli in human monocytic cells. TSP-1 production is upregulated through TLR2 signaling on the NF-κB-dependent pathway. Inflammatory cytokines such as IL-17F are involved in TSP-1 induction. In conclusion, the specific role of TSP-1 in periodontal disease is unknown, but it may be a potential target during the progression of periodontitis. Future studies are needed to elucidate the function of TSP-1 in periodontal disease.

## Supporting Information

S1 Table **Gene expression in THP-1 cells stimulated with** ***P. gingivalis*** **LPS by DNA microarray.** THP-1 cells were stimulated with 1.0 µg/ml of *P. gingivalis* LPS for 4 h. Total RNA from stimulated (Test) and non-stimulated (Control) cells were amplified. The *P. gingivalis* LPS-stimulated sample was labeled with Cy3 and the control sample with Cy5. The total data sheet shows the raw data, BG subtraction values, and global normalization values, including the Cy3/Cy5 ratio. In total, 25,369 genes were detected. Various genes were upregulated in THP-1 cells, of which TSP-1 expression was increased 14.5-fold.
(XLS)
