# Correction: Thrombospondin-1 Production Is Enhanced by *Porphyromonas gingivalis* Lipopolysaccharide in THP-1 Cells

**DOI:** 10.1371/journal.pone.0139759

**Published:** 2015-09-29

**Authors:** Misa Gokyu, Hiroaki Kobayashi, Hiromi Nanbara, Takeaki Sudo, Yuichi Ikeda, Tomonari Suda, Yuichi Izumi

In Fig 2, there is an error in the units of the X-axis of Fig 2C. Additionally, there is an error in the legend for Fig 2. Specifically, the time of incubation in Fig 2E is incorrect. Please view the complete, correct Fig 2 and figure legend here:

**Fig 2:**
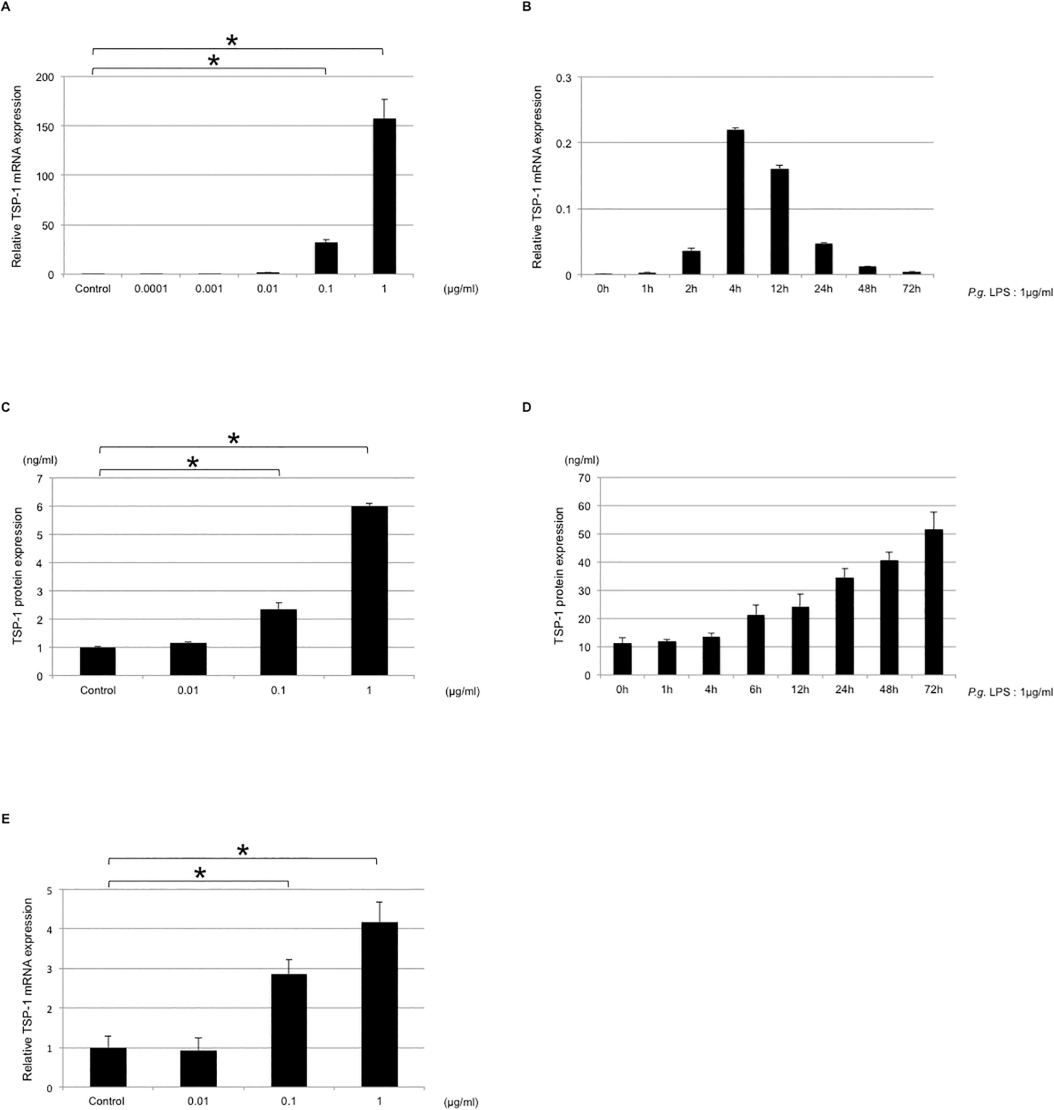
Upregulation of TSP-1 in THP-1 cells by P. *gingivalis* LPS stimulation. (A) THP-1 cells were stimulated by P. *gingivalis* LPS at concentrations of 0, 0.0001, 0.001, 0.01, 0.1, or 1.0 μg/ml for 4 h. P. gingivalis LPS increased TSP-1 mRNA expression in a dose-dependent manner in THP-1 cells. (B) THP-1 cells were stimulated by 1.0 µg/ml of P. *gingivalis* LPS for 0, 1, 2, 4, 12, 24, 48, or 72 h. P. *gingivalis* LPS increased TSP-1 mRNA expression in a time-dependent manner in THP-1 cells. (C) THP-1 cells were stimulated with P. *gingivalis* LPS at concentrations of 0, 0.01, 0.1, or 1.0 μg/ml for 72 h. P. *gingivalis* LPS increased TSP-1 protein production in a dose-dependent manner in THP-1 cells. (D) THP-1 cells were stimulated with 1.0 μg/ml of P. *gingivalis* LPS for 0, 1, 4, 6, 12, 24, 48, or 72 h. P. *gingivalis* LPS increased TSP-1 protein production in a time-dependent manner in THP-1 cells. (E) PMA-treated THP-1 cells were stimulated with P. gingivalis LPS at concentrations of 0, 0.01, 0.1, or 1.0 μg/ml for 4 h. P. *gingivalis* LPS increased TSP-1 protein production in a dose-dependent manner in PMA-treated THP-1 cells.
